# A Stochastic Petri Net-Based Model of the Involvement of Interleukin 18 in Atherosclerosis

**DOI:** 10.3390/ijms21228574

**Published:** 2020-11-13

**Authors:** Dorota Formanowicz, Agnieszka Rybarczyk, Marcin Radom, Krzysztof Tanaś, Piotr Formanowicz

**Affiliations:** 1 Department of Clinical Biochemistry and Laboratory Medicine, Poznan University of Medical Sciences, 60-806 Poznan, Poland; doforman@ump.edu.pl; 2 Institute of Computing Science, Poznan University of Technology, 60-965 Poznan, Poland; Agnieszka.Rybarczyk@cs.put.poznan.pl (A.R.); Marcin.Radom@cs.put.poznan.pl (M.R.); Krzysztof.Tanas@cs.put.poznan.pl (K.T.); 3 Institute of Bioorganic Chemistry, Polish Academy of Sciences, 61-704 Poznan, Poland; 4 Faculty of Electrical Engineering, Gdynia Maritime University, 81-225 Gdynia, Poland

**Keywords:** atherosclerosis, inflammation, mathematical modeling, Petri net, stochastic Petri net, t-invariants

## Abstract

Interleukin 18 (IL-18) is a proinflammatory and proatherogenic cytokine with pleiotropic properties, which is involved in T and NK cell maturation and the synthesis of other inflammatory cytokines and cell adhesion molecules. It plays a significant role in orchestrating the cytokine cascade, accelerates atherosclerosis and influences plaque vulnerability. To investigate the influence of IL-18 cytokine on atherosclerosis development, a stochastic Petri net model was built and then analyzed. First, MCT-sets and t-clusters were generated, then knockout and simulation-based analysis was conducted. The application of systems approach that was used in this research enabled an in-depth analysis of the studied phenomenon. Our results gave us better insight into the studied phenomenon and allow revealing that activation of macrophages by the classical pathway and IL-18-MyD88 signaling axis is crucial for the modeled process.

## 1. Introduction

There are many indications that one of the basic features of animate objects is their complexity. In other words, their nature results from the complicated structure of interactions between their constituent basic objects and processes. It means that for a real understanding of the structure and functioning of living organisms, a systems approach is necessary, consisting of the perception and study of organisms as systems, i.e., sets of independent, but interconnected and cooperating elements.

Systems are definitely more than the sum of their components. It is because a system has functionality that is not provided by a single subsystem or any of its components. It can be said that such a system cannot be simplified. To understand it, it is necessary to analyze it as a whole, because studying only its individual elements will not allow obtaining an accurate picture of such a system. The systems approach is therefore based on the analysis of the examined object as a whole, paying particular attention to all interactions taking place inside it, and at the same time not neglecting a detailed analysis of the basic elements of which it consists.

The dynamic development of systems biology and systems medicine observed in recent years is undoubtedly a consequence of the emergence of new opportunities both on the side of biological and medical sciences and technical sciences. On the one hand, they result from the creation of more and more accurate models of phenomena, resulting from advances on the basis of molecular biology, biochemistry and biophysics, on the other hand, they are the result of the increasingly better computing capabilities of modern computers and the use of more and more sophisticated methods of analyzing the obtained results. A systems approach to medicine may therefore turn out to be stimulating for many fields, including, for example, computer science or cybernetics, which so far have not been intuitively associated with medicine.

To apply a systems approach to the analysis of a given process, its model needs to be created, which should be as accurate as possible. The precision determines the correctness of the results from the model analysis. Such a model should be expressed in the language of some branch of mathematics. One of them is model types based on graph theory and related areas of mathematics. Among them are the models used in our study expressed in the language of Petri nets theory. These models allow describing the structure of the analyzed system precisely, hence their analysis may facilitate the understanding of its behavior. There are many extensions of the Petri net that can take into account different kinds of data. It is worth mentioning that such an extension usually does not change the structure of the model. Besides, there are many mathematical methods and software tools for analyzing and simulating Petri nets to support model analysis.

Atherosclerosis, in the context of the complexity and the knowledge about underlying pathways, is a process that is very well suited for systems research. This disorder is perceived as a complex inflammatory-fibroproliferative process taking place in the inner membrane of large and medium-sized arteries, the dynamics of which is continuously modified by many factors. The main morphological symptom of atherosclerosis is the appearance of foam cells in the intima of the arteries. In contrast, much more subtle changes, mainly caused by the influx of inflammatory cells and immunocompetent cells, and the local release of cytokines and other inflammatory mediators, occur in the microenvironment of the arterial wall [1]. Our motivation for this research was to investigate the complex interactions that affect interleukin 18 (IL-18) as well as the effects of the cytokine itself on related pathways in atherosclerosis. It should be noted here, that IL-18 was originally termed as IFN-γ-inducing factor derived from anti-CD3-stimulated T-helper cells. It is a unique cytokine that plays an important role in immune, inflammatory and infectious diseases. Not all of its actions are dependent on IFN-γ. In addition, IL-18 has been reported to play an important role in the progression and destabilization of the atherosclerotic plaque. Its unique features in health and disease in experimental animals and humans have been described in detail in [2]. Moreover, the latest knowledge about IL-18 and its significance for the human organism has been widely reviewed in [3]. To better understand the analyzed process we presented a flow chart along with an explanation of the most important subprocesses that we took into account. We created a model, which was merely presented in [4] to show a new approach to the analysis of this complex phenomenon. Since then, our analysis capabilities have evolved, which allowed us to conduct more advanced research on this model. Here, we presented the stochastic Petri net model of the involvement of IL-18 in the atherosclerosis process. To the best of our knowledge, studies using stochastic Petri nets to model the participation of IL-18 in the process of atherosclerotic plaque formation have not been conducted so far.

The organization of this paper is as follows. In Section 2 some basics of Petri nets theory useful for understanding the next parts of the paper, together with the informal description of the biological system are provided. In Section 3 the Petri net model is presented. This section contains also the results of the model analysis. The paper ends with conclusions in Section 4.

## 2. Methods

### 2.1. Petri Nets and Stochastic Petri Nets

The proposed model of the analyzed biological process is expressed in the language of Petri nets theory, hence in this subsection a brief introduction to the basics of this theory is given. Here the idea of Petri nets is explained and some notions important for the analysis of the proposed model are described.

A Petri net is a mathematical object whose structure is a weighted directed bipartite graph. Graph of this type consists of two disjoint subsets of vertices. In Petri nets these vertices are called places and transitions. They can be connected by arcs in such a way that only an arc connecting a place with a transition or a transition with a place is possible (i.e., no two places nor two transitions can be connected). In the biological context, places represent passive components of a modeled system such as chemical compounds while transitions correspond to its active components such as chemical reactions. The arcs, which model causal relationships between active and passive components of the system, are labeled with positive integer numbers called weights. However, a Petri net is not simply a graph that determines its structure (which should correspond to the structure of the modeled system). One of the fundamental properties of Petri nets is their dynamics which is brought to them by the components of another type, i.e., tokens. They reside in places and represent quantities of respective passive components of the system. The distribution of tokens over places is called marking and corresponds to a state of the modeled system [5,6,7].

More formally, a Petri net is 5-tuple $Q=(P,T,F,W,M_{0})$, where: $P=\{p_{1},p_{2},\ldots ,p_{n}\}$ is a finite set of places, $T=\{t_{1},t_{2},\ldots ,t_{m}\}$ is a finite set of transitions, F⊆(P×T)∪(T×P) is a set of arcs, $W:F\to Z^{+}$ is a weight function, $M_{0}:P\to N$ is an initial marking, P∩T=∅∧P∪T≠∅ [5].

Tokens flow from one place to another via transitions what corresponds to a flow of substances, information etc. through the modeled system. It is governed by the transition firing rule according to which a transition is enabled if each of its pre-places (i.e., the ones who are its immediate predecessors) contains several tokens equal to or greater than the weight of an arc connecting this place with the transition. An enabled transition can fire, meaning that the tokens can flow from its pre-places to its post-places (i.e., the ones being direct successors of the transition). The number of flowing tokens between the place and the transition is equal to the weights of an arc connecting these two vertices.

Petri nets have a very intuitive graphical representation, which is helpful in understanding the structure and behavior of the modeled system. In this representation places are depicted as circles, transitions as rectangles, arcs as arrows, tokens as dots or positive integer numbers residing in places and weights as positive integer numbers associated with arcs (if a weight of an arc is equal to 1, then it is not shown).

A stochastic Petri net (SPN) is an extension of a classical Petri net already described. An SPN description preserves the discrete numbers of tokens on its places, but in addition associates a probabilistically distributed firing rate (firing delay) with each transition. All actions which occur in a classical Petri net can still occur in an SPN, but their likelihood depends on the probability distribution of the associated firing rates. Formally, a stochastic Petri net is 6-tuple $Q_{S}=\left(\;P,T,F,W,M_{0},v\right)$, where $\left(\;P,T,F,W,M_{0}\right)$ is a marked classical Petri net underlying an SPN [8]. The list of marking dependent firing rates associated with transitions is denoted by $v=\{\lambda_{1},\lambda_{2},...,\lambda_{m}\}$. The firing rates are typically transition-specific and state-dependent. Transition firing time is exponentially distributed and the distribution of random variable $\chi_{j}$ of the firing time of transition $t_{j}$ is given by $f_{\chi_{j}}\left(\;\tau \right)\;=1-e^{-\lambda_{j}\tau },\;\tau \geq 0$ [9].

We assume that firing time is state-dependent, i.e., the number of tokens in pre-places of a transition influence that time as stated by mass action law. Such functions are taken into account and supported by popular tools, such as Snoopy [10]. If there is more than one enabled transition, a race for the next firing will take place and in averaged case, the transition with the smallest waiting time will fire next. Hence, in the case of an SPN the system behavior is described by the same discrete state space as in the case of a classical Petri net, and all the different sequences of events of a classical Petri net can still take place. Therefore, it allows to use the same analysis techniques for a stochastic Petri net as for a classical one [11].

It should be mentioned that there is more types of nets considered to be stochastic ones. The type of a stochastic Petri net described above is also called in the literature as a continuous-time SPN [9]. Another type of a stochastic net is for example a generalized SPN, where an additional, second type of transition is introduced. The so-called an immediate transition fires immediately when it is enabled. There is also possibility of adding some constraints to firing time resulting in other subtypes of an SPN, or using additional rules governing the flow of tokens, such as in a queueing Petri nets (also described in [9]). It is worth mentioning that there exist many other variants of Petri nets which can be useful in various applications (see, e.g., [6,7,12,13,14]). In our study the standard stochastic Petri net described in this section proved to be best suited for the analysis and simulation of the proposed model.

#### 2.1.1. t-Invariants

Despite that the graphical representation of a Petri net is intuitive and useful, it is not very well suited for an analysis of its mathematical properties. For this purpose a different representation, called incidence matrix, is used. In this matrix, $A={\left[a_{ij}\right]}_{n\times m}$, where *n* is the number of places and *m* is the number of transitions, rows correspond to places while columns correspond to transitions. Every entry $a_{ij}$, is an integer number equal to a difference between the numbers of tokens in place $p_{i}$ after and before firing transition $t_{j}$.

When a Petri net is a model of a biological system often especially important is an analysis of its invariants. There are two types of them, i.e., *place invariants* (*p-invariants*) and *transition invariants* (*t-invariants*). A p-invariant is a vector $y\in N^{n}$ being a solution of equation $A^{T}\cdot y=0$. A t-invariant is a vector $x\in N^{m}$ satisfying equation A·x=0.

In this work we will focus on the analysis of t-invariants. With each such an invariant *x* there is associated its *support*, denoted by supp(x). A support supp(x) of t-invariant *x* is a set of transitions which correspond to positive entries of *x*, i.e., $supp\left(x\right)=\left\{t_{j}:x_{j}>0,j=1,2,\ldots ,m\right\}$. t-invariant *x* is *minimal* if its support does not properly contain a support of any other t-invariant, i.e., if there is no t-invariant $x^{\prime }$ such that $supp\left(x^{\prime }\right)\subset supp\left(x\right)$. A Petri net is *covered* by t-invariants if every transition belongs to support of at least one t-invariant. An important property of t-invariants is that they correspond to subprocesses which do not change the state of a modeled system.

#### 2.1.2. t-Clusters and MCT Sets

In the analysis based on t-invariants often important are similarities among them. When the number of such invariants is large, they can be grouped into structures called *t-clusters* using standard clustrering algorithms. In general, clustering of t-invariants is neither a simple nor an automatic process since there are plenty of clustering algorithms and similarity measures. To determine the best joining algorithm, an appropriate number of clusters and a suitable similarity measure we conducted a computational experiment in which various clusterings (i.e., the sets of clusters) have been generated. To evaluate them and select the best one with the optimal number of t-clusters, we used Mean Split Silhouette (MSS) [15] and Calinski-Harabasz (C-H) [16] evaluation indexes. After comparing multiple clusterings, similarly as in [17], we decided to base our analysis on the clusters obtained using Unweighted Pair Group Method with Arithmetic Mean (UPGMA) algorithm and the correlated Pearson similarity measure, which seem to provide usually the best t-clusters in case of a Petri net analysis [18]. Every t-cluster corresponds to some functional block of the analyzed biological system, so we determined biological meanings of all the obtained clusters.

Similarly like t-invariants, which are grouped into t-clusters, also transitions can be grouped into the so-called *MCT sets* (*Maximal Common Transition sets*) [19,20]. A set of this type consists of transitions which belong to supports of exactly the same t-invariants. MCT sets partition the set of transitions into disjoint subsets corresponding to functional modules of a biological system. t-invariants and MCT sets are basis for conducting detailed analysis of the system [21]. It is easy to see that a support of every t-invariant is a collection of some MCT sets (some of them may contain only one element and are called trivial MCT sets).

The relations between MCT sets can also be studied. Many non-trivial MCT sets are close to each other within the net that they share places which either provide or store tokens produced by transitions belonging to the specific sets. A simple example of such relation can be one place which store tokens necessary for two distinct sets (i.e., transitions belonging to two MCT sets). This is an example of a conflict between two sets. One can also imagine a situation, when a transition from one set produce tokens necessary to activate a transition of a different set. Such relations have been studied and are given in a Table S1.

#### 2.1.3. Knockout Analysis

Another type of analysis of Petri net models of biological systems is based on knockout approach, which has been previously presented in [17,22]. It allows to investigate which subprocesses will be influenced if some selected elementary functional unit is disabled.

In general, knockout analysis can be based on t-invariants (a so-called *t-invariant-based knockout*) or on the net simulation (*simulation knockout*) [17]. In the case of the t-invariant-based knockout some chosen transitions are excluded from the system and the remaining t-invariants, which then may not fully cover the net, are examined. What follows, the transitions that are not covered are considered disabled, because they are dependent on the ones that have been initially knocked out. It is worth noting that it is very interesting from the biological point of view to identify which parts of the net will be affected by the knockout of the selected transitions and also which transitions should be knocked out to achieve a desired behavior of the model. The second type of such an analysis is a simulation knockout which involves the net simulation and an analysis of tokens distribution in places in a situation when some selected transitions have been knocked out in the model. For this purpose stochastic Petri nets are especially well suited, especially when mass action law can be considered during such a simulation. By disabling a transition we mean that it never fires during a simulation. Then, a series of simulations are performed, all starting from the same initial marking and ending after achieving the same number of steps. In such a way an influence of a knockout of some important reactions (transitions) on the rest of the model can be studied.

**Figure 1:**
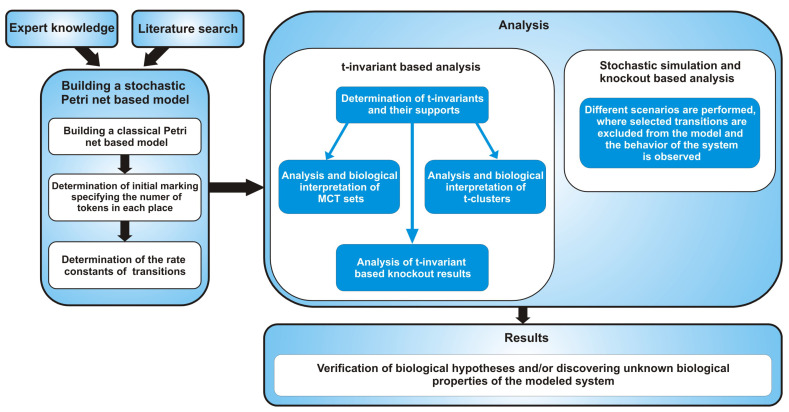
The general flow chart comprising the steps starting from building a stochastic Petri net-based model, through various methods of its analysis to obtaining results.

The above mentioned consecutive stages of the process of creating and analysis of a stochastic Petri net model are shown in Figure 1.

### 2.2. The Involvement of Interleukin 18 in Atherosclerosis Related Phenomena that Was Taken into Account for Building the Model

This subsection presents the modeled disorder, divided into thematic blocks together with their detailed description and features, along with the corresponding place and transition symbols that are included in the model. Both places and transitions are denoted by the notation $p_{a}$ and $t_{b}$, where *a* and *b* correspond to their respective numbers from Table 1 and Table 2.

**Figure 2:**
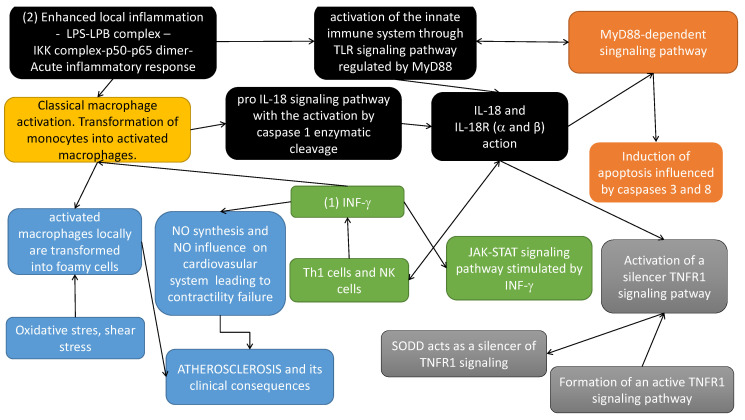
Dependencies in the modeled system

To better understand the modeled issue, the most important dependencies of the modeled process have been presented in Figure 2.

1. (yellow box in Figure 2) Classical macrophage activation occurs in response to the action of: (1) IFN-γ (originally called macrophage-activating factor) and (2) lipopolysaccharide (LPS) (enhanced (severe) local inflammation-LPS-LPB complex-IKK complex phosphorylated and initiation cascade of phosphorylation leading finally to the translocation of p50–p65 to the nucleus and gene transcription of many inflammatory cytokines that activate inflammatory cascade).
2. (black boxes in Figure 2) IL-18 is a proinflammatory cytokine, expressed mainly by activated macrophages. This cytokine facilitates IFN-γ production mainly by Th1 cells and NK (natural killers) cells. Induction of cellular immunity modulates acute phase reaction, in the model by affecting IL-18 receptors (α and β) action. It should be noted that IL-18 is synthesized as an inactive precursor (pro IL-18) requiring processing by caspase 1 into an active form of cytokine (pro IL-18 signalling pathway with the activation by caspase 1 enzymatic cleavage). Caspase 1 is activated within inflammasomes. Finally, activated IL-18 acts by binding to its receptors (α and β). Different types of macrophages (M1 and M2) are involved in its regulation [2].
3. (blue boxes in Figure 2) Activated macrophages, influenced locally by enhanced oxidative stress, inflammatory process and shear stress, increase the atherosclerotic plaque formation, leading along with local changes of nitric oxide (NO), to the atherosclerosis-based clinical consequences.
4. (green boxes in Figure 2) IFN-γ induces transcription of several proinflammatory genes, for example, inducible NO synthase (iNOS). IFN-γ signal transduction pathway starts with the recruitment of Janus kinases (JAK1 and JAK2), to the IFN-γ receptor inducing their phosphorylation in tyrosine. As a consequence, the transcription factor STAT1α is recruited to the IFN-γR and phosphorylated by JAKs. Phosphorylated Stat1α dimerizes and translocates to the nucleus. Here it induces transcriptional activation of several genes by binding to the proper sites of their promoters. Among the genes induced, IFN regulatory factor (IRF) 1, is also transcription factor which mediates the transcriptional regulation induced by IFN-γ [34].
5. (orange boxes in Figure 2) The cytoplasmic TIR domains of the IL-18R complex interact with myeloid differentiation factor 88 (MyD88). IL-18 together with MyD88 mediates apoptosis via extrinsic and intrinsic pathways. It up-regulates Fas, FasL, TNFR1 and induces TNF synthesis. The extrinsic pathways evolves the recruitment of FADD to Fas/FasL and TRADD to TNF/TNFR1, which activates caspase 8 and caspase 3 action [35].
6. (gray boxes in Figure 2) TNFR pathway. TLR and TNFR superfamily members can cooperate to regulate cell death and inflammation generated by pathogen ligands [3].

## 3. Results and Discussion

### 3.1. Building a Stochastic Petri Net Based Model

Here, a stochastic Petri net-based model of the involvement of the IL-18 cytokine in the formation of the atherosclerotic plaque in patients suffering from CKD is presented. The model is based on a classical Petri net introduced in [4] and extended in [2] to investigate the dependencies between Il-18 and different types of macrophages and the different pathways of IL-18 synthesis. In both cases the analysis was based on t-invariants.

**Figure 3:**
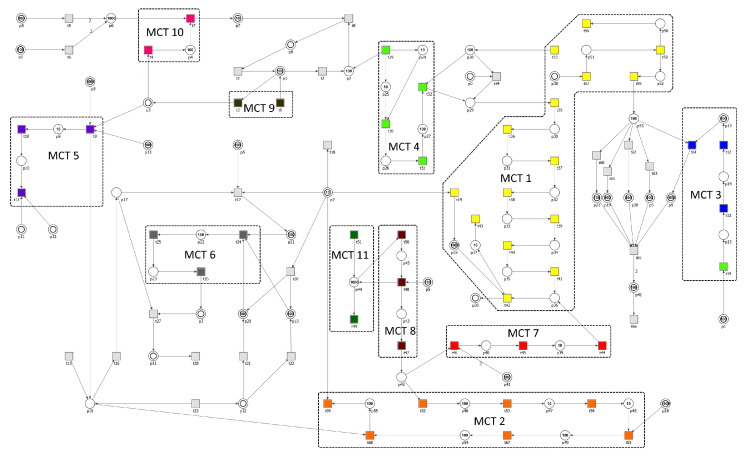
The Petri net-based model with initial marking and MCT sets marked by accordingly labeled rectangles. The transitions within a given MCT set are encoded in the same color. Logical places are depicted as two concentric circles. Places and transitions are represented by their numbers.

The considered stochastic model consists of 56 places and 70 transitions, which names and biological meaning is described in Table 1 and Table 2, respectively. The graphical representation of the net, together with initial marking and names of places and transitions, is presented in Figure 3. The initial marking specifying the number of tokens in each place is presented in Table 3 and was assigned basing on [36,37,38]. Some places in the model are shown as two concentric circles. They graphically represent the same place in the net and are called logical places (e.g., place $p_{18}$ has 3 different locations). Their role is to simplify the visualization of connections between the nodes in the net and to improve its readability.

The net presented in [4] is qualitative what means that it does not contain any information about time dependencies between various components of the system and it describes only its structure. Such time dependencies are present in real processes and are often crucial for understanding their nature. Although they are not known precisely, they can be estimated by durations of biochemical reactions in the system and their reaction rates. Such extension makes the studied system more realistic and is included in the extended model shown in this paper. All stochastic transitions in the model follow mass-action kinetics and the kinetic parameters (rate constants) were calculated according to the simple heuristic developed in [38]. Since it is more biologically intuitive, we first assigned six different time constants (i.e., durations) to the reactions (i.e., transitions), which are listed in Table 4. To determine those values, first, we identified the processes in the model that have the shortest and the longest time duration. Next, based on the literature and expert knowledge, we elaborated a time scale with values ranging from 1 to 1000 (see Table 4). Finally, we calculated the rate constants of the stochastic transitions as a reciprocal of those values. The obtained rate constants are presented in Table 5.

### 3.2. The Analysis Based on T-Invariants

The analysis of clusters, MCT sets and the formal properties of the model has been done using our recently published tool Holmes [43]. First, let us mention some basic structural properties of the net. The net is pure (there are no loops, i.e., read arcs), not ordinary (there are four arcs weighted with two) and connected, but not in the strong sense. Moreover, it is also not structurally conflict-free (there are transitions with common pre-places) and is unbounded, since there is no upper bound on the number of tokens. The model contains neither input nor output places, but there are several input and output transitions.

#### 3.2.1. The Analysis and Biological Interpretation of MCT Sets

The model contains 223 minimal t-invariants and the net is covered by them. Based on them, MCT sets have been generated. There are 11 non-trivial MCT sets (i.e., sets containing more than one transition) and they are listed in Table 6 together with their biological interpretation. All of them represent connected subnetworks and they divide the structure of the net into disjoint regions.

#### 3.2.2. The Analysis and Biological Interpretation of T-Clusters

Next, we grouped t-invariants into t-clusters. To obtain biologically relevant ones, we used UPGMA (Unweighted Pair Group Method with Arithmetic Mean) clustering algorithm and correlated Pearson as a similarity measure. The resultant clusters are listed and described in Table 7. One of these clusters is very large, while the others are rather small. From this, it follows that process of atherosclerotic plaque formation is very complex. There are many independent signaling pathways modeled within the analyzed system, but they are all essential to the creation and development of atherosclerosis.

#### 3.2.3. The Analysis of t-Invariants Based Knockout Results

Next, in order to get a deeper insight into the system behavior we decided to conduct a t-invariants based knockout analysis. First, we decided to investigate the importance of each MCT set (both trivial i.e., single transitions and non-trivial ones) within the analyzed model. To achieve this, an in silico knockout analysis with the use of MonaLisa software [44] and our newly published tool Holmes [43], based on t-invariants has been performed [22,45]. Here, in case of each transition knocked out, other transitions that are affected by such a knockout have been computed (transitions belonging to the same non-trivial MCT set operate together thus their knockout impact is the same). The affected transitions are those, that are present in the support of affected t-invariants. The results are shown in Table 8 and they indicate that the most critical to the tested system are the following pathways: activation of macrophages, formation of the IL-18-IL-18Rs complex together with pro IL-18 signaling pathway, MyD88-dependent signaling, formation of the TNFR1 signaling complex and JAK/STAT pathway stimulated by INF-γ. All of them are associated with atherosclerosis development and progression.

As it has been stated before, t-invariant is a specific subprocess, while its support defines the basic reactions (transitions). If at least one reaction is disabled, then the whole process cannot accomplish its task. One can be particularly interested in knowing what influences two specific transitions in the net: $t_{28}$ (atherosclerotic plaque) and $t_{11}$ (cardiovascular disease). Firing any one of them depends on tokens in $p_{11}$ (foamy cells), while $t_{11}$ additionally requires tokens in $p_{12}$ (high shear stress). Production of tokens in $p_{11}$ depends on the following pathway (arrows denote production of tokens in a given place by a specific transition): $p_{11}\leftarrow t_{27}$ (transformation into foamy cells) $\leftarrow p_{17}$ (modified oxidized LDL) $\leftarrow t_{16}$ (lipids peroxydation). It should be noted that $t_{27}$ also requires tokens from $p_{3}$ (activated macrophages), however stopping the production in this place results in disabling all t-invariants except for one (222 out of 223 total).

If $t_{16}$ is disabled by any reason, only 70 t-invariants remain, none of which contain neither $t_{11}$ nor $t_{28}$. As a result 7 transitions are not covered by the remaining t-invariants:

- pathway $t_{9}$ (NO synthesis) $\to t_{10}$ (cardiac contractile dysfunction) $\to t_{11}$ (cardiovascular disease),
- pathway $t_{16}$ (lipids peroxydation) $\to t_{27}$ (transformation into foamy cells) $\to t_{28}$ (atherosclerotic plaque),
- $t_{17}$ (neighboring endothelial cells stimulation) required for tokens in $p_{21}$ (MCP1) which is not a serious problem because for production of tokens in $p_{21}$ is also responsible transition $t_{60}$ (MCP1 gene transcription).

To disable $t_{16}$ one needs to disable its source of tokens in $p_{16}$ (peroxynitrite). Two transitions produce tokens there: $t_{15}$ (reaction catalysed by NADPH oxydase) and $t_{23}$ (hemodialysis). Disabling only $t_{15}$ has only light consequences: 194 t-invariants remain and the net is still fully covered. Unfortunately, only one t-invariant with $t_{28}$ in the support is also disabled, while all t-invariants with $t_{11}$ still remain. However, if $t_{23}$ is also disabled, only 30 t-invariants remain and apart from disabling 7 aforementioned transitions we eliminate whole MCT set number 2 (responsible for induction of apoptosis influenced by caspases 3 and 8, that are situated at pivotal junctions in apoptosis pathways).

One can now ask a question if it is at least possible to reduce the number of processes (t-invariants) responsible for activation $t_{11}$ and $t_{28}$, when disabling them completely, as describe above, bears too serious consequences for the net processes. The normal state of the net when nothing is disabled is as follows: 223 t-invariants, 32 with $t_{11}$ (cardiovascular disease) in their supports and 53 with $t_{28}$ (atherosclerotic plaque). Four t-invariants contain both studied transitions in their supports. Several scenarios have been tested and analysed.

- Disabling of $t_{60}$ (MCP1 gene transcription) results in a net covered (completely) by only 127 t-invariants, $t_{11}:32\backslash 32$ and $t_{28}:17\backslash 53$, which means that none t-invariant with $t_{11}$ is disabled in the process (32 out of 32 remain) while only 17 t-invariants — 32% with $t_{28}$ remain as a result.
- Disabling of $t_{61}$ (VCAM1 transcription): 151 t-invariants remain, net is fully covered, $t_{11}:20\backslash 32$ and $t_{28}:41\backslash 53$.
- Disabling of $t_{62}$ (ICAM1 transcription): same as for $t_{61}$.
- Disabling of $t_{64}$ (iNOS gene transcription): 71 t-invariants remain, net is not covered, $t_{11}:0\backslash 32$ and $t_{28}:17\backslash 53$. As a result this scenario disables: MCT3, MCT7 and single transitions $t_{65}$ (colorredproinflammatory response), $t_{66}$ (no apoptosis) and the already mentioned path $t_{9}\to t_{10}\to t_{11}$.
- Disabling of $t_{63}$ (TNF gene transcription): 22 t-invariants remain, net is not covered, $t_{11}:3\backslash 32$ and $t_{28}:13\backslash 53$. As a result this scenario disables: MCT2, MCT7, MCT8 and single transitions $t_{5}$ (modulation by TNF), $t_{17}$ (neighboring endothelial cells stimulation), $t_{65}$ (proinflammatory response) and $t_{66}$ (no apoptosis).

Two last combined scenarios aimed to minimize the number of active processes containing $t_{11}$ and $t_{28}$ are given below.

- Disabling $t_{64}$ with $t_{61}$ or $t_{62}$ (or both of them): 51 t-invariants remain, the net is still fully covered, $t_{11}:0\backslash 32$ and $t_{28}:13\backslash 53$. Disabled transitions and MCT sets are the same as described in scenario for disabling $t_{64}$ alone.
- Disabling $t_{60}$ (MCP1 gene transcription) and $t_{64}$ (iNOS gene transcription): 39 t-invariants remain, the net is not covered, $t_{11}:0\backslash 32$ and $t_{28}:5\backslash 53$, so only 5 processed resulting in atherosclerotic plaque remain.

### 3.3. Stochastic Simulation and Knockout Based Analysis

To be able to answer some important biological questions, we carried out a simulation based analysis of the system using Gillespie stochastic simulation algorithm (SSA) [46] available in Snoopy [10]. In each simulation experiment, the algorithm has been run 50,000 times, where each run consisted of 1,000,000 steps. Additionally, we conducted simulation knockout of the model. Here, selected transitions were excluded from the model, in our case by assigning to them a firing rate equal to 0 and the behavior of the system was examined. In stochastic simulation, we observed the selected places: $p_{9}$ (NO), $p_{10}$ (foamy cells), $p_{53}$ (p50–p65 dimer in the nucleus).

**Scenario** **1.** *The influence of the IL-18 and IFN-* γ *on the atherosclerosis development and progression*

**Scenario** **1A.** *The influence of the IL-18 cytokine on the atherosclerosis development*

In this simulation experiment, we decided to inspect the role of IL-18 in the development of cardiovascular disease and atherosclerosis progression. Evidence shows, that IL-18 is a proinflammatory and proatherogenic cytokine with pleotropic properties, involved in T and NK cells maturation, production of other inflammatory cytokines and cell adhesion molecules. It plays a significant role in orchestrating the cytokine cascade and accelerates atherosclerosis and plaque vulnerability [47]. It was also demonstrated that IL-18 cytokine is as a good diagnostic marker of the acute inflammatory process, an index of myocardial necrosis and in the pathophysiology of atherosclerosis [4].

**Figure 4:**
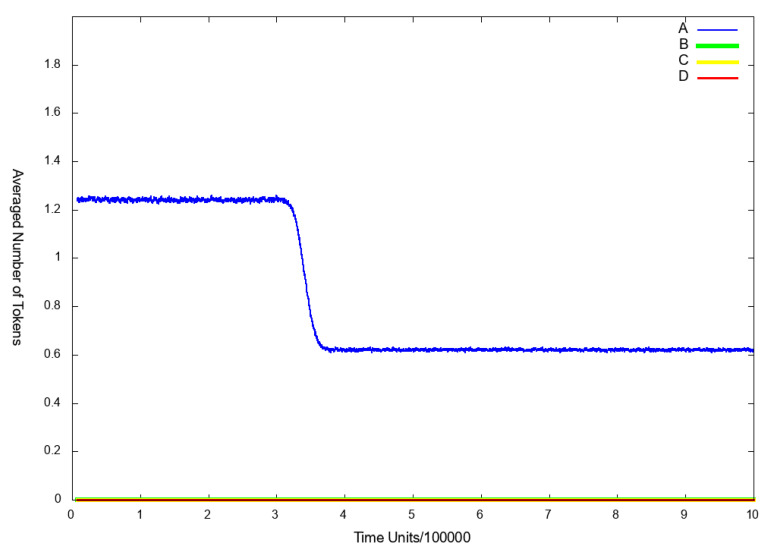
The results of in silico knockout analysis for place $p_{9}$ (nitric oxide NO). (**A**) The simulation of the model without knockout. (**B**) The impact of knocking out transitions $t_{7}$ (pro IL-18 activation by caspase 1 enzymatic cleavage) and $t_{0}$ (induction cell mediated immunity) (**C**) The impact of knocking out transitions $t_{0}$ (induction cell mediated immunity) and $t_{1}$ (INF-γ synthesis influencing) (**D**) The impact of knocking out transitions $t_{20}$ (ICAM1 and VCAM1 up regulation), $t_{23}$ (hemodialysis (HD)), $t_{61}$ (VCAM1 transcription) and $t_{62}$ (ICAM1 transcription) and $t_{0}$ (induction cell-mediated immunity).

To observe the influence of IL-18 on the analyzed system, we excluded from the model the following transitions: $t_{7}$ (pro IL-18 activation by caspase 1 enzymatic cleavage) and $t_{0}$ (induction cell mediated immunity). Firstly, we decided to observe the place $p_{9}$ (nitric oxide (NO)), since it reflects the risk of atherosclerosis related cardiovascular events, which are connected with higher risk of death. As it can be seen in Figure 4B, if IL-18 is inhibited, cardiovascular disease does not develop.

**Figure 5:**
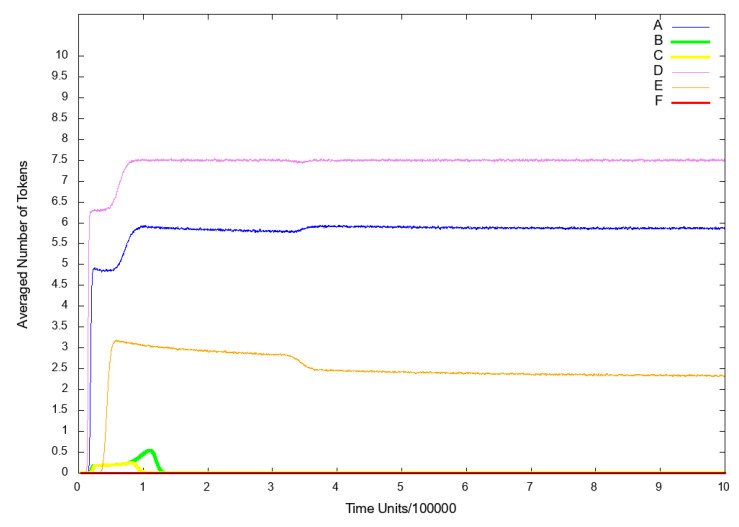
The results of in silico knockout analysis for place $p_{10}$ (foamy cells). (A) The simulation of the model without knockout. (**B**) The impact of knocking out transitions $t_{7}$ (pro IL-18 activation by caspase 1 enzymatic cleavage) and $t_{0}$ (induction cell mediated immunity) (**C**) The impact of knocking out transitions $t_{0}$ (induction cell-mediated immunity) and $t_{1}$ (INF-γ synthesis influencing) (**D**) The impact of knocking out transition $t_{42}$ (IKK complex phosphorylation) (**E**) The impact of knocking out transitions $t_{20}$ (ICAM1 and VCAM1 up regulation), $t_{23}$ (HD), $t_{61}$ (VCAM1 transcription) and $t_{62}$ (ICAM1 transcription) (**F**) The impact of knocking out transitions $t_{20}$ (ICAM1 and VCAM1 up regulation), $t_{23}$ (HD), $t_{61}$ (VCAM1 transcription) and $t_{62}$ (ICAM1 transcription) and $t_{0}$ (induction cell mediated immunity).

Additionally, we also observed place $p_{10}$ (foamy cells) as it represents the changes connected with atherosclerosis development. The results are presented in Figure 5 (line B) and it can be easily noticed, that the inhibition of IL-18 synergistically causes the inhibition of foamy cells formation and thereby halt atherosclerotic progression.

**Figure 6:**
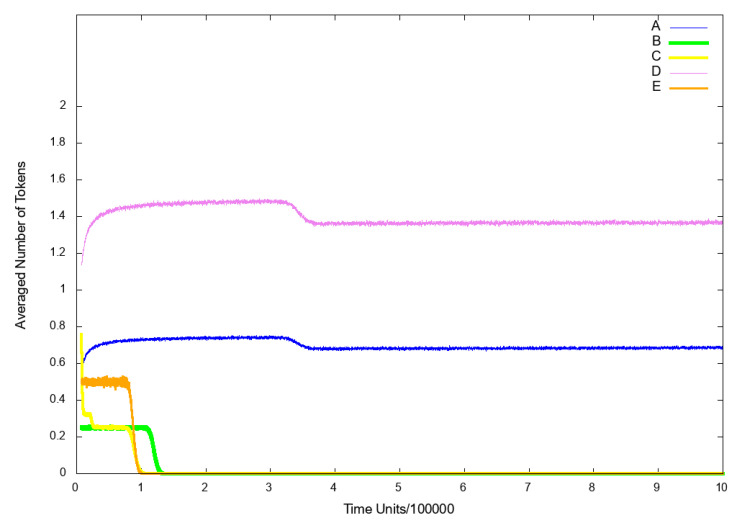
The results of in silico knockout analysis for place $p_{53}$ (p50–p65 dimer in the nucleus). (**A**) The simulation of the model without knockout. (**B**) The impact of knocking out transitions $t_{7}$ (pro IL-18 activation by caspase 1 enzymatic cleavage) and $t_{0}$ (induction cell mediated immunity) (**C**) The impact of knocking out transitions $t_{0}$ (induction cell mediated immunity) and $t_{1}$ (INF-γ synthesis influencing) (**D**) The impact of knocking out transitions $t_{20}$ (ICAM1 and VCAM1 up regulation), $t_{23}$ (HD), $t_{61}$ (VCAM1 transcription) and $t_{62}$ (ICAM1 transcription) (**E**) The impact of knocking out transitions $t_{20}$ (ICAM1 and VCAM1 up regulation), $t_{23}$ (HD), $t_{61}$ (VCAM1 transcription) and $t_{62}$ (ICAM1 transcription) and $t_{0}$ (induction cell-mediated immunity).

Another place, that we decided to observe was $p_{53}$ (p50–p65 dimer in the nucleus), because the translocation of activated NF-κB complexes from the cytoplasm to the nucleus is required to promote transcription of pro-inflammatory and pro-atherogenic genes. As it can be seen in Figure 6 (line B), NF-κB is not transported to the nucleus, hence the NF-κB-dependent transcription is strongly attenuated.

These findings are consistent with the existing literature [4,47].

**Scenario** **1B.** *The influence of the INF-γ on the atherosclerosis development*

INF-γ is a pro-inflammatory cytokine, which is secreted by T lymphocytes and macrophages. It is involved in the initiation and modulation of a variety of immune responses and is expressed at high levels in atherosclerotic lesions. It is also capable of influencing several key steps during atherosclerosis development. This includes pro-inflammatory gene expression, the recruitment of monocytes from the blood to the activated endothelium and plaque stability [47,48].

To analyze the influence of the INF-γ on the system, we excluded from the net the following transitions: $t_{0}$ (induction cell mediated immunity) and $t_{1}$ (INF-γ synthesis influencing). We have decided to observe the following places: $p_{9}$ (NO), $p_{10}$ (foamy cells) and $p_{53}$ (p50–p65 dimer in the nucleus) for the same reasons set out in Scenario 1A above. As a consequence, similarly as in case of Scenario 1A, inhibition of INF-γ resulted in a strong decrease in atherosclerotic plaque formation (see Figure 5 (line C)) and blockade of NF-κB transcription factor activation (see Figure 6 (line C)).

These findings are consistent with the existing literature [47,48].

**Scenario** **2.** *The influence of the inhibition of NF-* κ *B activation on the atherosclerosis development*

Atherosclerosis and inflammatory diseases involve activation of NF-κB, which is now considered to be a one of the key transcription factor and also a critical regulator of immune homeostasis. However, the molecular mechanisms by which NF-κB activation exerts beneficial or pathogenic signaling remain largely elusive [33,49].

To examine the influence of the NF-κB on the model, we excluded from the net the transition $t_{42}$ (IKK complex phosphorylation). In the similar way operates known NF-κB inhibitor i.e., pyrrolidine dithiocarbamate (PDTC) [50]. Next, we decided to look at the place $p_{10}$ (foamy cells) for the same reasons set out in Scenario Section 3.3 above. As a result, we could observe that the level of foamy cells has increased (see Figure 5 (line D)) as compared to the model without the above mentioned transition being knocked out (see Figure 5 (line A)). These results are consistent with the existing literature [49,51] and suggest that a certain level of NF-κB is necessary to modulate and counteract pro-atherogenic and inflammatory responses. Before one will be able to design safe and effective therapeutic approaches using NF-κB inhibitors, it is first necessary to sufficiently understand complex and often opposing functions of this transcription factor both on the molecular and cellular level [49].

**Scenario** **3.** *The influence of blocking agents targeting cell adhesion molecules on the atherosclerosis development and progression*

**Scenario** **3A.** *Influence of the inhibition of endothelial adhesion molecules VCAM1 and ICAM1 activation on the atherosclerosis development*

An early phase of atherosclerosis involves the recruitment of leukocytes, which is mainly mediated by cellular adhesion molecules (i.e., VCAM1, ICAM1) that are expressed on the vascular endothelium. The information obtained from human and animals models study suggests that those molecules are important players in the atherogenic process and play a crucial role in the atherosclerosis development and plaque instability. It has also been shown that independently of other inflammatory markers VCAM1 and ICAM1 were identified as a strong predictors of future cardiovascular events [52]. Unfortunately, relatively little attention has been paid so far to the therapeutic potential of vascular cell adhesion molecules involved in the development or progression of atherosclerosis although they seem to be a promising inflammatory disease targets [53].

To analyze the influence of the endothelial adhesion molecules VCAM1 and ICAM1 on the system, we excluded from the net the following transitions: $t_{20}$ (ICAM1 and VCAM1 up regulation), $t_{23}$ (HD), $t_{61}$ (VCAM1 transcription) and $t_{62}$ (ICAM1 transcription). We decided to observe two places: $p_{10}$ (foamy cells) and $p_{53}$ (p50–p65 dimer in the nucleus) for the same reasons set out in Scenario 1A above. As it can be seen, the level of p50–p65 dimer translocated to the nucleus has increased, which suggests that a NF-κB-dependent inflammation is up-regulated (see Figure 6 (line D) in comparison to model without knockout presented in Figure 6 (line A)). Nevertheless, the level of foamy cells has significantly decreased (see Figure 5 (line E)) as compared with the results shown in Figure 5 (line A) (the model without knockout). This result is consistent with the findings in the existing literature, where it was demonstrated that blocking adhesion molecules such as VCAM1 ameliorates atherosclerosis in apolipoprotein E-deficient mice [53].

**Scenario** **3B.** *Influence of the inhibition of early inflammation process and endothelial adhesion molecules VCAM1 and ICAM1 activation on the atherosclerosis development.*

If in addition to transitions knocked out in Scenario 3A, transition $t_{0}$ (induction cell mediated immunity) is deactivated, the NF-κB complexes are not transported to the nucleus and the expression of inflammatory genes dependent on it, is inhibited (see Figure 6 (line E)). Furthermore, foamy cells formation is strongly inhibited and what follows atherosclerosis progression is attenuated (see Figure 5 (line F)) and also the cardiovascular disease does not develop (see Figure 4 (line D)).

It is worth noting that the same effect can be achieved by the knockout of the following transitions: $t_{0}$ (induction cell mediated immunity), $t_{20}$ (ICAM1 and VCAM1 up regulation), $t_{23}$ (HD) and $t_{42}$ (IKK complex phosphorylation).

## 4. Conclusions

The results of our research show beyond any doubt that in atherosclerosis, being immuno-inflammatory disorder, IL-18 and INF-γ play a key role. Moreover, deactivation of the NF-κB pathway in the model increases the development of inflammation underlying atherosclerosis and its clinical consequences. In addition, the inhibition of the participation of (1) adhesive molecules (ICAM1 and VCAM1), (2) cell-mediated immunity, (3) the effect of dialysis treatment (HD) and (4) IKK complex phosphorylation significantly reduces the development of atherosclerotic plaque. The formation of atherosclerotic plaque still takes place but to a lesser extent. Our results show how harmful could be dialysis in the context of the studied phenomenon. In addition, we showed that in order for the modeled process to occur, an activation of macrophages by the classical pathway and IL-18-MyD88 signaling axis is required. Moreover, concerning the modulation by IFN-γ, we have shown that although blocking this elementary process itself does not affect other elementary processes (transitions), it has an impact on almost three-fourths of subprocesses (t-invariants) in the modeled atherosclerosis phenomenon. This is important because it should be noted that IFN-γ in the human body acts by modulating the immune response to infection. So, it seems that in the case of atherosclerosis, the body’s response to damaging factors, including, for example, bacterial infections, is crucial. Hence, it is an additional confirmation of the inflammatory basis underlying the development of atherosclerotic plaque. Additionally, we proved that IL-18, together with the directly dependent signaling pathways is crucial for atherosclerosis, and can be considered a good candidate for a biomarker of atherosclerosis.

## Figures and Tables

**Table 1:** The list of places of the model.

Place	Biological Meaning	References	Place	Biological Meaning	References
$p_{0}$	active IL-18-IL-18Rs (α and β) complex	[3,23]	$p_{28}$	MyD88	[24]
$p_{1}$	INF-γ synthesized by T and NK cells	[3,23,25]	$p_{29}$	Receptors MyD88 complex	[24]
$p_{2}$	IL-18R	[3,23]	$p_{30}$	TLR-MyD88-IRAK4 complex	[24]
$p_{3}$	activated macrophages	[3,23,26]	$p_{31}$	TLR-MyD88-IRAK4 -IRAK1P complex	[24]
$p_{4}$	pro IL-18	[3]	$p_{32}$	TLR-MyD88-IRAK4-IRAK1P-TRAF6 complex	[24]
$p_{5}$	TNF	[3,26,27,28,29]	$p_{33}$	IRAK1P-TRAF6-TAB2-TAB1-TAK1 complex at plasma membrane	[24]
$p_{6}$	ICE	[3]	$p_{34}$	IRAK1 degraded	[24]
$p_{7}$	IL-18	[3]	$p_{35}$	UBC13-UEV1A-TRAF6-TAK1P-TAB2P-TAB1 complex	[24]
$p_{8}$	iNOS activated	[28,30]	$p_{36}$	TAK1 activated	[24]
$p_{9}$	NO	[30]	$p_{37}$	MAP kinase phosphorylation activation	[28]
$p_{10}$	contractility failure	[30]	$p_{38}$	IKK complex phosphorylated activated	[24,28]
$p_{11}$	foamy cells	[26,30]	$p_{39}$	RIP1 ubiquitinated	[29]
$p_{12}$	high shear stress	[30,31]	$p_{40}$	TRAF2 autoubiquitinated	[29]
$p_{13}$	IFR1	[25]	$p_{41}$	FLIP	[28,29]
$p_{14}$	JAK-STAT pathway activated	[23,25]	$p_{42}$	TRAF2-TRADD-RIP1-TRAF5-TNFR complex	[29]
$p_{15}$	INF-γ-IFNRs complex	[23,25]	$p_{43}$	active TNFR1 signalling complex	[29]
$p_{16}$	peroxynitrite	[30,31]	$p_{44}$	SODD	[27]
$p_{17}$	modified oxidized LDL	[26,30]	$p_{45}$	TNFR1 stable with SODD	[27]
$p_{18}$	FASL and FAS	[3,29]	$p_{46}$	TNFR1 endocytosed	[29]
$p_{19}$	VCAM1	[3,26,28,30]	$p_{47}$	FADD recruited	[29]
$p_{20}$	ICAM1	[3,26,28,30]	$p_{48}$	pro caspase 8 recruited	[29]
$p_{21}$	MCP1	[26,28]	$p_{49}$	DISC	[29]
$p_{22}$	monocytes	[26]	$p_{50}$	IκB phosphorylated	[28]
$p_{23}$	macrophages	[26]	$p_{51}$	p50–p65-IκB phosporylated complex	[28]
$p_{24}$	LPS binding protein LBP	[32]	$p_{52}$	p50–p65 dimer-NFκB (early phase)	[28]
$p_{25}$	microbial infection LPS	[32]	$p_{53}$	p50–p65 dimer in the nucleus	[28]
$p_{26}$	LPS-LPB complex	[32]	$p_{54}$	caspase 8 active	[29]
$p_{27}$	TL4 activated	[32]	$p_{55}$	caspase 3 active	[29,31]

**Table 2:** The list of transitions of the model.

Transition	Biological Meaning	References	Transition	Biological Meaning	References
$t_{0}$	induction cell-mediated immunity	[23]	$t_{35}$	IRAK4 recruitment	[24]
$t_{1}$	INF-γ synthesis influencing	[23]	$t_{36}$	connection with IRAK1 and its phosphorylation	[24]
$t_{2}$	infection modulation	[23]	$t_{37}$	TRAF6 recruitment and binding to IRAK1	[24]
$t_{3}$	classical macrophages activation by INF-γ	[3,23]	$t_{38}$	IRAK1P-TRAF6 dissociation	[24]
$t_{4}$	pro IL-18 synthesis	[3]	$t_{39}$	TAK1-TAB2 phosphorylation	[24]
$t_{5}$	modulation by TNF	[3]	$t_{40}$	TRAF6-TAK1P-TAB2P-TAB1 complex translocation to cytosol	[24]
$t_{6}$	modulation by INF-γ	[3,23]	$t_{41}$	TRAF6 ubiquitination	[24]
$t_{7}$	pro IL-18 activation by caspase 1 enzymatic cleavage	[3]	$t_{42}$	IKK complex phosphorylation	[24,28,33]
$t_{8}$	IL-18 and IL-18Rs binding	[3]	$t_{43}$	p38 and MAPK signalling pathway	[28]
$t_{9}$	nitric oxide synthesis	[30]	$t_{44}$	RIP1 recruites TAK1 via TAB2	[29]
$t_{10}$	cardiac contractile dysfunction	[30]	$t_{45}$	RIP1 ubiquitination	[29]
$t_{11}$	cardiovascular disease (CVD)	[30]	$t_{46}$	TRAF2 ubiquitination	[29]
$t_{12}$	IFR1 synthesis	[25]	$t_{47}$	forming TRAF2-TRADD- RIP1-TRAF5 complex	[29]
$t_{13}$	JAK and STAT pathway activation	[23,25]	$t_{48}$	TNFR1 trimerization	[27,29]
$t_{14}$	INF-γ-IFNRs interaction	[23,25]	$t_{49}$	usage	[27]
$t_{15}$	reaction catalysed by NADPH oxydase	[26,30]	$t_{50}$	TNFR1 stabilization	[27]
$t_{16}$	lipids peroxydation	[26,30]	$t_{51}$	SODD expression	[27]
$t_{17}$	neighboring endothelial cells stimulation	[3,26]	$t_{52}$	TNFR1 endocytosis	[29]
$t_{18}$	neovascularization inhibition	[3]	$t_{53}$	FADD recruitment	[29]
$t_{19}$	FasL expression on inflammatory cells	[3]	$t_{54}$	pro caspase 8 recruitment	[29]
$t_{20}$	ICAM1 and VCAM1 up regulation	[3]	$t_{55}$	connection through DDs	[29]
$t_{21}$	ICAM1 increasing	[30]	$t_{56}$	ubiquitylation and degradation	[28,33]
$t_{22}$	VCAM1 decreasing	[30]	$t_{57}$	phosphorylation cascade initiation	[28,33]
$t_{23}$	hemodialysis (HD)	[31]	$t_{58}$	IκB dissociation from the complex	[28,33]
$t_{24}$	attracting monocytes	[26]	$t_{59}$	p50–p65 translocation to the nucleus	[28,33]
$t_{25}$	transformation into macrophages	[26]	$t_{60}$	MCP1 gene transcription	[28]
$t_{26}$	activation	[26]	$t_{61}$	VCAM1 transcription	[26,28]
$t_{27}$	transformation into foamy cells	[26,30]	$t_{62}$	ICAM1 transcription	[28]
$t_{28}$	atherosclerotic plaque	[26]	$t_{63}$	TNF gene transcription	[28,29]
$t_{29}$	severe inflammation	[3,32]	$t_{64}$	iNOS gene transcription	[28,30]
$t_{30}$	LPS and LBP binding	[32]	$t_{65}$	proinflammatory response	[28,29,30]
$t_{31}$	LPS presentation to TLR4 and CD14	[32]	$t_{66}$	no apoptosis	[28,29]
$t_{32}$	TLR4 and MyD88 connection	[24]	$t_{67}$	caspase 8 autocleavage and activation	[29]
$t_{33}$	MyD88 recruitment	[24]	$t_{68}$	caspase 3 activation	[29,31]
$t_{34}$	active IL-18-IL-18Rs (α and β) and MyD88 connection	[3]	$t_{69}$	apoptosis enhancement	[29,31]

**Table 3:** List of places and the initial numbers of tokens. We specified and assigned to places three different values approximating the actual concentration of the molecules, complexes and receptors in the model [36,37,38]. Those values reflect the following relationships: 1000—high concentration, 100—medium concentration, 100—low concentration, 0—very low concentration or absence. The concentrations are given in units of $pM=10^{-12}M$.

Place	Initial Number of Tokens	Place	Initial Number of Tokens	Place	Initial Number of Tokens	Place	Initial Number of Tokens
$p_{0}$	0	$p_{14}$	0	$p_{28}$	100	$p_{42}$	0
$p_{1}$	100	$p_{15}$	0	$p_{29}$	0	$p_{43}$	0
$p_{2}$	100	$p_{16}$	0	$p_{30}$	0	$p_{44}$	1000
$p_{3}$	0	$p_{17}$	0	$p_{31}$	0	$p_{45}$	0
$p_{4}$	100	$p_{18}$	1000	$p_{32}$	0	$p_{46}$	100
$p_{5}$	100	$p_{19}$	100	$p_{33}$	0	$p_{47}$	10
$p_{6}$	1000	$p_{20}$	100	$p_{34}$	0	$p_{48}$	10
$p_{7}$	10	$p_{21}$	100	$p_{35}$	0	$p_{49}$	100
$p_{8}$	1000	$p_{22}$	100	$p_{36}$	0	$p_{50}$	0
$p_{9}$	10	$p_{23}$	0	$p_{37}$	10	$p_{51}$	0
$p_{10}$	0	$p_{24}$	10	$p_{38}$	0	$p_{52}$	0
$p_{11}$	0	$p_{25}$	10	$p_{39}$	10	$p_{53}$	100
$p_{12}$	0	$p_{26}$	0	$p_{40}$	0	$p_{54}$	100
$p_{13}$	100	$p_{27}$	100	$p_{41}$	100	$p_{55}$	100

**Table 4:** The specification of the durations and time intervals assigned to the components of the model. Unfortunately, in the case of the modeled processes (see column ’Process’) only approximate time values can be found in the literature (see columns ’Time Information Coming from the Available Literature’ and ’Time Interval’). Thus, we recalculated those values using the scale of 1 to 1000 (see column ’Duration’) to model the time dependencies between them.

Process	Duration	Time Information Coming from the Available Literature	Time Interval
binding of ligands and proteins, influence of molecules, activation of enzymes, proteins and processes	1	seconds [39]	Very Short
recruitment, connection, phosphorylation, stabilization of molecules, modulation of the activation of proteins and processes, ubiquitination	40	seconds to a minute [40]	Short
decrease in molecules availability, dissociation of protein complexes	100	about twice shorter than synthesis and expression [41]	Medium
synthesis of biomolecules (e.g., INF-γ, pro IL-18), increase in molecules availability/production, TNFR1 endocytosis, translocation of protein complexes from cytosol to nucleus and from nucleus to cytosol, lipids peroxidation	200	>minutes [40]	Long
severe inflammation, differentiation of monocytes into macrophages	500	>hours [37]	Very Long
atherosclerotic plaque development, CVD	1000	>days [37,41]	Very, Very Long

**Table 5:** The list of the rate functions for the analyzed model. MA(c) denotes the mass action function [42] and *c* is a kinetic parameter (rate constant), which is presented in the units of $sec^{-1}$. MA is a predefined function, available in Snoopy [10] that creates the rate function for a given transition out of its input places and takes a kinetic parameter as argument.

Transition	Kinetic Parameter *c*	Rate Function	Transition	Kinetic Parameter *c*	Rate Function
$t_{0}$	1.0	MA(1.0)	$t_{35}$	0.025	MA(0.025)
$t_{1}$	1.0	MA(1.0)	$t_{36}$	0.025	MA(0.025)
$t_{2}$	0.01	MA(0.01)	$t_{37}$	0.025	MA(0.025)
$t_{3}$	1.0	MA(1.0)	$t_{38}$	0.01	MA(0.01)
$t_{4}$	0.005	MA(0.005)	$t_{39}$	0.025	MA(0.025)
$t_{5}$	1.0	MA(1.0)	$t_{40}$	0.005	MA(0.005)
$t_{6}$	1.0	MA(1.0)	$t_{41}$	0.025	MA(0.025)
$t_{7}$	1.0	MA(1.0)	$t_{42}$	0.025	MA(0.025)
$t_{8}$	1.0	MA(1.0)	$t_{43}$	0.005	MA(0.005)
$t_{9}$	0.005	MA(0.005)	$t_{44}$	0.025	MA(0.025)
$t_{10}$	0.002	MA(0.002)	$t_{45}$	0.025	MA(0.025)
$t_{11}$	0.002	MA(0.002)	$t_{46}$	0.025	MA(0.025)
$t_{12}$	0.005	MA(0.005)	$t_{47}$	0.005	MA(0.005)
$t_{13}$	1.0	MA(1.0)	$t_{48}$	0.005	MA(0.005)
$t_{14}$	0.005	MA(0.005)	$t_{49}$	0.002	MA(0.002)
$t_{15}$	0.01	MA(0.01)	$t_{50}$	0.025	MA(0.025)
$t_{16}$	0.005	MA(0.005)	$t_{51}$	0.005	MA(0.005)
$t_{17}$	0.005	MA(0.005)	$t_{52}$	0.005	MA(0.005)
$t_{18}$	0.005	MA(0.005)	$t_{53}$	0.025	MA(0.025)
$t_{19}$	0.005	MA(0.005)	$t_{54}$	0.025	MA(0.025)
$t_{20}$	0.025	MA(0.025)	$t_{55}$	0.025	MA(0.025)
$t_{21}$	0.005	MA(0.005)	$t_{56}$	0.005	MA(0.005)
$t_{22}$	0.01	MA(0.01)	$t_{57}$	1.0	MA(1.0)
$t_{23}$	0.005	MA(0.005)	$t_{58}$	0.01	MA(0.01)
$t_{24}$	0.025	MA(0.025)	$t_{59}$	0.005	MA(0.005)
$t_{25}$	0.005	MA(0.005)	$t_{60}$	0.005	MA(0.005)
$t_{26}$	1.0	MA(1.0)	$t_{61}$	0.005	MA(0.005)
$t_{27}$	0.005	MA(0.005)	$t_{62}$	0.005	MA(0.005)
$t_{28}$	0.002	MA(0.002)	$t_{63}$	0.005	MA(0.005)
$t_{29}$	0.005	MA(0.005)	$t_{64}$	0.005	MA(0.005)
$t_{30}$	1.0	MA(1.0)	$t_{65}$	0.025	MA(0.025)
$t_{31}$	0.025	MA(0.025)	$t_{66}$	0.005	MA(0.005)
$t_{32}$	0.025	MA(0.025)	$t_{67}$	0.025	MA(0.025)
$t_{33}$	0.025	MA(0.025)	$t_{68}$	1.0	MA(1.0)
$t_{34}$	0.025	MA(0.025)	$t_{69}$	0.002	MA(0.002)

**Table 6:** The list of non-trivial MCT sets.

MCT-Set	Contained Transitions	Biological Interpretation
$m_{1}$	$t_{19}$, $t_{33}$, $t_{35}$, $t_{36}$, $t_{37}$, $t_{38}$, $t_{39}$, $t_{40}$, $t_{41}$, $t_{42}$, $t_{43}$, $t_{56}$, $t_{57}$, $t_{58}$, $t_{59}$	MyD88-dependent signaling pathway
$m_{2}$	$t_{52}$, $t_{53}$, $t_{54}$, $t_{55}$, $t_{67}$, $t_{68}$, $t_{69}$	induction of apoptosis influenced by caspases 3 and 8 that are situated at pivotal junctions in apoptosis pathways
$m_{3}$	$t_{12}$, $t_{13}$, $t_{14}$, $t_{64}$	JAK-STAT signaling pathway stimulated by INF-γ and its impact on the regulation of iNOS expression
$m_{4}$	$t_{29}$, $t_{30}$, $t_{31}$, $t_{32}$	activation of the innate immune system through TLR signaling pathways regulated by TIR domain-containing adaptors, such as MyD88
$m_{5}$	$t_{9}$, $t_{10}$, $t_{11}$	influence of nitric oxide on cardiovascular system
$m_{6}$	$t_{24}$, $t_{25}$, $t_{26}$	transformation of monocytes into activated macrophages
$m_{7}$	$t_{44}$, $t_{45}$, $t_{46}$	activation of a silencer of TNFR1 signaling pathway
$m_{8}$	$t_{47}$, $t_{48}$, $t_{50}$	formation of an active TNFR1 signaling complex
$m_{9}$	$t_{0}$, $t_{3}$	activation of macrophages by the classical pathway
$m_{10}$	$t_{4}$, $t_{7}$	pro IL-18 signaling pathway
$m_{11}$	$t_{49}$, $t_{51}$	SODD signaling pathway

**Table 7:** The 223 minimal *t*-invariants of the model clustered by UPGMA algorithm. The second and third column of the table give the total number of *t*-invariants in the cluster, together with its biological interpretation. The last column lists the processes contained in the clusters.

Cluster No.	Biological Interpretation	No. of t-Invariants	Contained Processes	
			MCT-Sets	Single Transitions
$c_{1}$	SODD signaling pathway	1	$m_{11}$	
$c_{2}$	The involvement of the IL-18 cytokine in the formation of the atherosclerotic plaque in patients suffering from CKD without influence of SODD signaling pathway	216	$m_{1}$, $m_{2}$, $m_{3}$, $m_{4}$, $m_{5}$, $m_{6}$, $m_{7}$, $m_{8}$, $m_{9}$, $m_{10}$	$t_{1}$, $t_{2}$, $t_{5}$, $t_{6}$, $t_{8}$, $t_{15}$, $t_{16}$, $t_{17}$, $t_{18}$, $t_{20}$, $t_{21}$, $t_{22}$, $t_{23}$, $t_{27}$, $t_{28}$, $t_{34}$, $t_{60}$, $t_{61}$, $t_{62}$, $t_{63}$, $t_{65}$, $t_{66}$
$c_{3}$	pro IL-18 signaling pathway and activation of macrophages by the classical pathway	1	$m_{9}$, $m_{10}$	$t_{6}$, $t_{18}$
$c_{4}$	IL-18 synthesis and IL-18- mediated INF-γ induction	1	$m_{9}$, $m_{10}$	$t_{1}$, $t_{2}$, $t_{6}$, $t_{8}$
$c_{5}$	activation of macrophages by INF-γ leading to lipids peroxidation and atherosclerotic plaque development	1	$m_{9}$	$t_{15}$, $t_{16}$, $t_{27}$, $t_{28}$
$c_{6}$	influence of NO on cardiovascular system and activation of the innate immune system through TLR together with pro IL-18 signaling pathway leading to atherosclerosis development	3	$m_{1}$, $m_{3}$, $m_{4}$, $m_{5}$, $m_{9}$, $m_{10}$	$t_{1}$, $t_{2}$, $t_{6}$, $t_{8}$, $t_{16}$, $t_{23}$, $t_{27}$, $t_{28}$, $t_{34}$

**Table 8:** The most important activities in the model according to their combinatorial knockout impact calculated based on the approach described in [17,22].

MCT Set	Activity	Knockout Impact (Transitions)	Knockout Impact (t-Invariants)
$m_{9}$	activation of macrophages by the classical pathway	95.71%	99.55%
$m_{10}$	pro-IL-18 signaling pathway	85.71%	99.10%
$t_{8}$	IL-18 and IL-18Rs binding	80.00%	98.65%
$m_{1}$	MyD88-dependent signaling pathway	75.71%	98.21%
$t_{63}$	TNF gene transcription	24.29%	90.13%
$t_{6}$	modulation by INF-γ	0%	73.54%
$t_{23}$	hemodialysis (HD)	7.14%	73.54%
$m_{4}$	activation of the innate immune system through TLR signaling pathways regulated by TIR domain-containing adaptors, such as MyD88	4.29%	73.54%
$t_{34}$	active IL-18-IL-18Rs (α and β) and MyD88 connection	0%	73.54%
$t_{16}$	lipids peroxydation	8.57%	68.61%
$m_{3}$	JAK-STAT signaling pathway stimulated by INF-γ and its impact on the regulation of iNOS expression	14.29%	68.16%
$t_{65}$	proinflammatory response	5.71%	60.99%
$m_{8}$	formation of an active TNFR1 signaling complex	17.14%	48.43%
$t_{17}$	neighboring endothelial cells stimulation	0%	48.43%
$t_{21}$	ICAM1 increasing	0%	43.05%
$t_{22}$	VCAM1 decreasing	0%	43.05%
$t_{60}$	MCP1 gene transcription	0%	43.05%
$t_{27}$	transformation into foamy cells	5.71%	36.32%
$t_{61}$	VCAM1 transcription	0%	32.29%
$t_{62}$	ICAM1 transcription	0%	32.29%
$m_{7}$	activation of a silencer of TNFR1 signaling pathway	2.86%	30.49%
$m_{6}$	transformation of monocytes into activated macrophages	2.86%	30.49%
$t_{66}$	no apoptosis	0%	30.49%
$t_{5}$	modulation by TNF	0%	25.56%
$m_{2}$	induction of apoptosis influenced by caspases 3 and 8 that are situated at pivotal junctions in apoptosis pathways	8.57%	25.11%
$t_{2}$	infection modulation	0%	25.11%
$t_{1}$	INF-γ synthesis influencing	0%	25.11%
$t_{28}$	atherosclerotic plaque	0%	23.77%
$t_{20}$	ICAM1 and VCAM1 up regulation	0%	21.52%
$m_{5}$	influence of NO on cardiovascular system	2.86%	14.35%
$t_{15}$	reaction catalysed by NADPH oxydase	0%	13.00%
$t_{18}$	neovascularization inhibition	0%	0.90%
$m_{11}$	SODD signaling pathway	1.43%	0.45%

## Supplementary Materials

The following are available at https://www.mdpi.com/1422-0067/21/22/8574/s1, Table S1: Interactions between MCT sets. In the second column some specic places for each given set are listed. For those places tokens are either provided by other net elements (outside of a given MCT set) or consumed by other net elements, effectively inhibiting function of a given MCT set.

## Abbreviations

The following abbreviations are used in this manuscript:

CD	cluster of differentiation
CKD	chronic kidney disease
DDs	Death Domains
DISC	death-inducing signaling complex
Fas	type I membrane protein that belongs to the tumor necrosis factor-nerve growth factor receptor family
FasL	Fas ligand (tumour necrosis factor superfamily)
FADD	Fas-associated protein with Death Domain
FLIPP	FADD-like-interleukin β converting enzyme-like inhibitory protein
HD	haemodialysis
ICAM	intercellular adhesion molecule
ICE	caspase1-protease
IFR	interferon-related protein
IKK	IκB kinase
IL-18	interleukin 18
IL-18Rs	Interleukin 18 receptors
INF-γ	interferon gamma
iNOS	inducible isoform nitric oxide synthase
IRAK	interleukin 1 receptor-associated kinase
IRAKP	interleukin 1 receptor-associated kinase phosphorylated
JAK/STAT	Janus kinase/signal transducers and activators of transcription
LBP	lipopolysaccharide binding protein
LPS	lipopolysaccharide
MAPK	mitogen-activated protein kinases
MCP1	monocyte chemoattractant protein 1
MCT sets	Maximal Common Transition sets
MPS	mononuclear phagocyte system
MSS	Mean Split Silhouette
MyD88	myeloid differentiation primary response 88
NADPH oxidase	nicotinamide adenine dinucleotide phosphate-oxidase
NK	natural killers
NO	nitric oxide
PN	Petri net
p38-MAPK	p38 mitogen-activated protein kinases
p50	nuclear factor kappa-B p50 subunit
p65	nuclear factor-kappa-B p65 subunit
RIP	receptor-interacting protein
SODD	silencer of death domains
SPN	stochastic Petri net
TAB	transforming growth factor β activated kinase
TAK	transforming growth factor β activated kinase-associated binding protein
TLR	Toll-like receptor
TNFR1	tumor necrosis factor receptor 1
TRADD	tumor necrosis factor receptor type 1—associated DEATH domain protein
TRAF	tumor necrosis factor receptor-associated factor
Uev1A-Ubc13	ubiquitin-conjugating enzyme complex
VCAM	vascular cell adhesion molecule
